# Virus-like particles as robust tools for functional assessment: Deciphering the pathogenicity of *ABCA4* genetic variants of uncertain significance

**DOI:** 10.1016/j.jbc.2024.107739

**Published:** 2024-08-31

**Authors:** Senem Cevik, Subhasis B. Biswas, Arit Ghosh, Esther E. Biswas-Fiss

**Affiliations:** 1 Department of Medical and Molecular Sciences, College of Health Sciences, University of Delaware, Newark, Delaware, USA; 2 Ammon Pinizzotto Biopharmaceutical Innovation Center, University of Delaware, Newark, Delaware, USA; 3 Delaware Biotechnology Institute, UD Center for Bioimaging, University of Delaware, Newark, Delaware, USA

**Keywords:** ABCA4, ABC transporter, membrane protein, retinal degeneration, human genetics, genetic disease, virus-like particles (VLPs), variants of uncertain significance (VUS), pathogenicity prediction, precision medicine

## Abstract

The retina-specific ABCA transporter, ABCA4, is essential for vision, and its genetic variants are associated with a wide range of inherited retinal degenerative diseases, leading to blindness. Of the 1630 identified missense variants in *ABCA4*, ∼50% are of unknown pathogenicity (variants of unknown significance, VUS). This genetic uncertainty presents three main challenges: (i) inability to predict disease-causing variants in relatives of inherited retinal degenerative disease patients with multiple *ABCA4* mutations; (ii) limitations in developing variant-specific treatments; and (iii) difficulty in using these variants for future disease prediction, affecting patients' life-planning and clinical trial participation. To unravel the clinical significance of *ABCA4* genetic variants at the level of protein function, we have developed a virus-like particle–based system that expresses the ABCA4 protein and its variants. We validated the efficacy of this system in the enzymatic characterization (ATPase activity) of VLPs harboring ABCA4 and two variants of established pathogenicity: p.N965S and p.C1488R. Our results were consistent with previous reports and clinical phenotypes. We also applied this platform to characterize the VUS p.Y1779F and observed a functional impairment, suggesting a potential pathogenic impact. This approach offers an efficient, high-throughput method for ABCA4 VUS characterization. Our research points to the significant promise of the VLP-based system in the functional analysis of membrane proteins, offering important perspectives on the disease-causing potential of genetic variants and shedding light on genetic conditions involving such proteins.

The retina-specific ABC transporter, ABCA4, has been established as an integral component of rod and cone cell disc membranes and is indispensable for vision, as it removes toxic byproducts generated through phototransduction from the photoreceptor cells in the retina. Variants in the *ABCA4* gene have been linked to a broad spectrum of inherited retinal degenerations, including Stargardt macular dystrophy (OMIM #248200) (1, 2, 3, 4, 5, 6, 7, 8, 9) fundus flavimaculatus (10, 11, 12, 13), autosomal recessive retinitis pigmentosa (OMIM #601718) (8, 13, 14, 15, 16, 17, 18), cone-rod dystrophy (OMIM #604116) (15, 19, 20, 21, 22, 23, 24), and potentially contribute to age-related macular degeneration (1, 2, 19, 25, 26, 27, 28). These disorders are characterized by progressive vision loss, and atrophy of the photoreceptors and retinal pigment epithelium, ultimately leading to blindness (29, 30). Stargardt disease is the leading cause of childhood blindness, affecting roughly 1:8000 to 1:10,000 individuals worldwide (31, 32, 33). As of this writing, 4043 genetic variants have been identified in the *ABCA4* gene, and 1630 of these have been identified as missense (https://www.ncbi.nlm.nih.gov/clinvar/ (accessed on April 2, 2024)).

Despite ongoing efforts to categorize variants into pathogenic/likely pathogenic and benign/likely benign classes, nearly 50% of *ABCA4*’s missense variants remain unclassified (variants of uncertain significance, VUS) or have conflicting interpretations of their clinical significance (11%) in ClinVar, (34, 35, 36, 37). While current computational approaches for predicting variant pathogenicity show promise by offering insights into complex biological processes, they fall short of meeting the growing demand for accurate predictions as stand-alone methods. The structural and functional consequences of the vast majority of these variants remain unknown (38, 39, 40, 41). Understanding the effect of genetic variations on disease and providing accurate risk assessment information from integrated approaches incorporating functional information is critical to the therapeutic assessment of patients (42, 43).

The American College of Medical Genetics-Association for Molecular Pathology standards regard well-established *in vitro* functional assessment of genetic variants as strong evidence to assess pathogenicity (44). Despite success in our and other laboratories (2, 4, 10, 21, 45, 46, 47, 48, 49, 50, 51, 52, 53, 54, 55, 56, 57), extensive functional characterization of *ABCA4* variants has proved difficult due to the challenges associated with working with a 2273 amino acid membrane protein and the number of variants now exceeding 4000. Traditional methods of purifying and characterizing membrane proteins involve detergent solubilization from membranes and, for the most part, reconstitution into artificial lipid environments. These steps are time-consuming, prone to protein denaturation, and may disrupt the native conformation and interactions of the protein (58, 59). Hence, the options for high-throughput functional characterization of large membrane proteins remain somewhat limited, particularly those that entail detergent solubilization of recombinantly expressed transporters followed by reconstitution in proteo-liposomes or nanodiscs. Thus, alternative approaches that simplify the expression and characterization of ABCA4 are essential to predict its genetic variants' molecular and functional consequences.

In this study, we aimed to establish an efficient virus-like particle (VLP)-based platform for the high-throughput functional characterization of a transmembrane protein, ABCA4, and its genetic variants. We illustrate that ABCA4 expressed in VLPs exhibited native topology, maintained functional activity, and appeared suitable for investigating protein behavior using a diverse range of biochemical and biophysical techniques without the necessity of lipid reconstitution (Fig. 1). We present a comprehensive overview of this successful approach, emphasizing its potential for high-throughput production of membrane proteins and the functional analysis of their disease-associated genetic variants.

**Figure 1:**
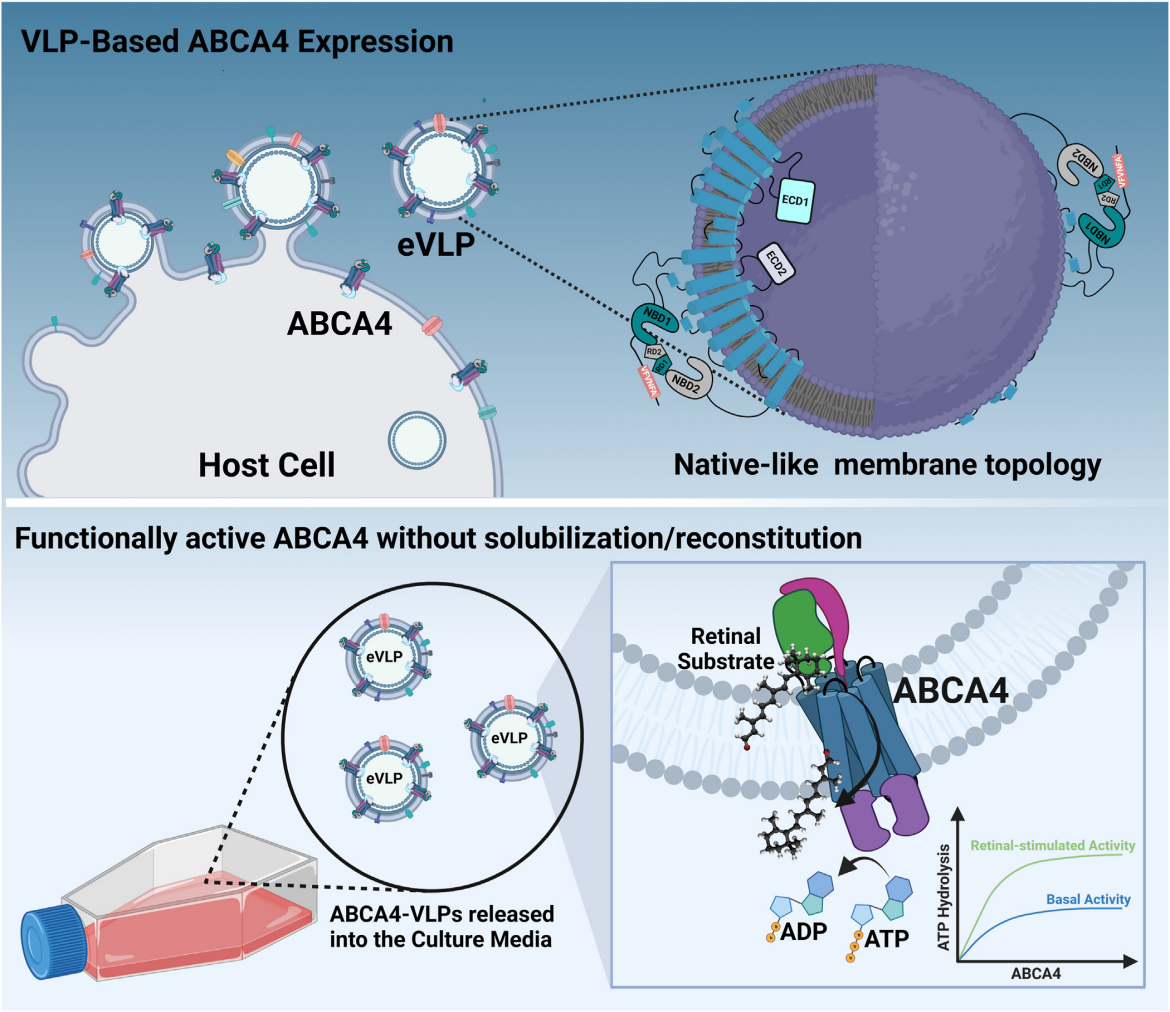
**Schematic representation of ABCA4 protein expression using a virus-like particle system.** ABCA4 is assembled into the membrane of VLPs within a host cell, mimicking its native membrane topology. Upon release into the culture media, the ABCA4-VLPs maintain their functional activity, enabling studies on ATP hydrolysis and interaction with the retinal substrate without the need for lipid reconstitution. This method facilitates the analysis of ABCA4’s function and the impact of genetic variations relevant to retinal diseases (created with BioRender.com).

## Results

### VLPs as a vehicle for recombinant membrane proteins

**Figure 2:**
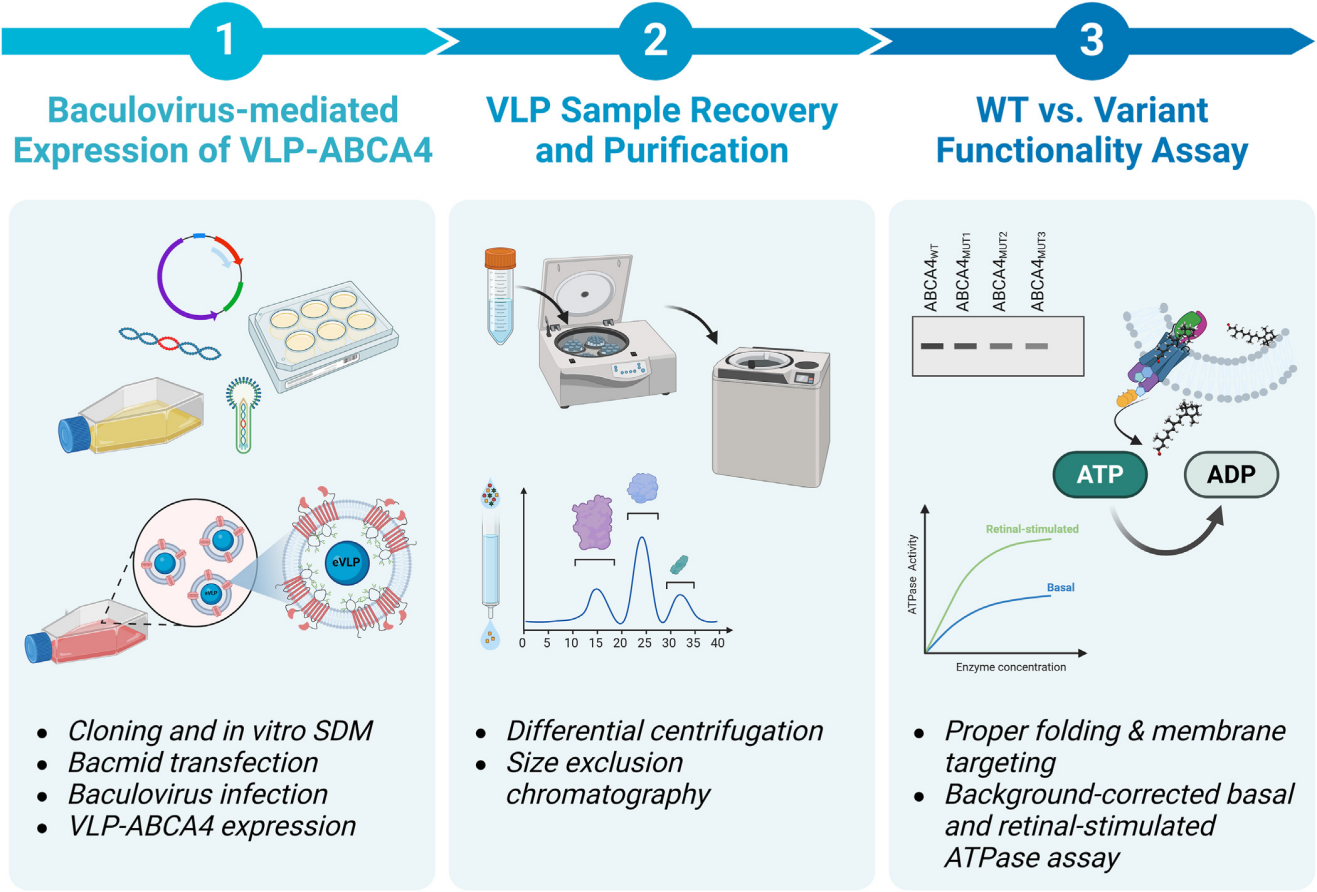
**Experimental workflow of the study.** Overview of the three key elements of the virus-like particle (VLP) platform, demonstrating its potential as a valuable tool in the functional study of membrane proteins and the characterization of their disease-associated variants (created with BioRender.com).

We have produced enveloped VLPs by coexpressing a membrane protein of interest and a structural viral core protein (in this instance, influenza virus matrix proteins) in a baculovirus expression vector system (BEVS). Unlike other sources of membrane proteins extracted from cell membranes, VLPs are readily available in cell culture media, homogeneous, and physically well-defined and present high concentrations of target membrane proteins in their native structure (60, 61). Our results demonstrate the efficacy of VLP-based expression platform in elucidating the functional impact of ABCA4 variants. An overview of the experimental platform's design is presented in Figure 2.

### Expression and purification of ABCA4 in enveloped VLPs

#### Expression of full-length ABCA4 in insect cells and VLPs

**Figure 3:**
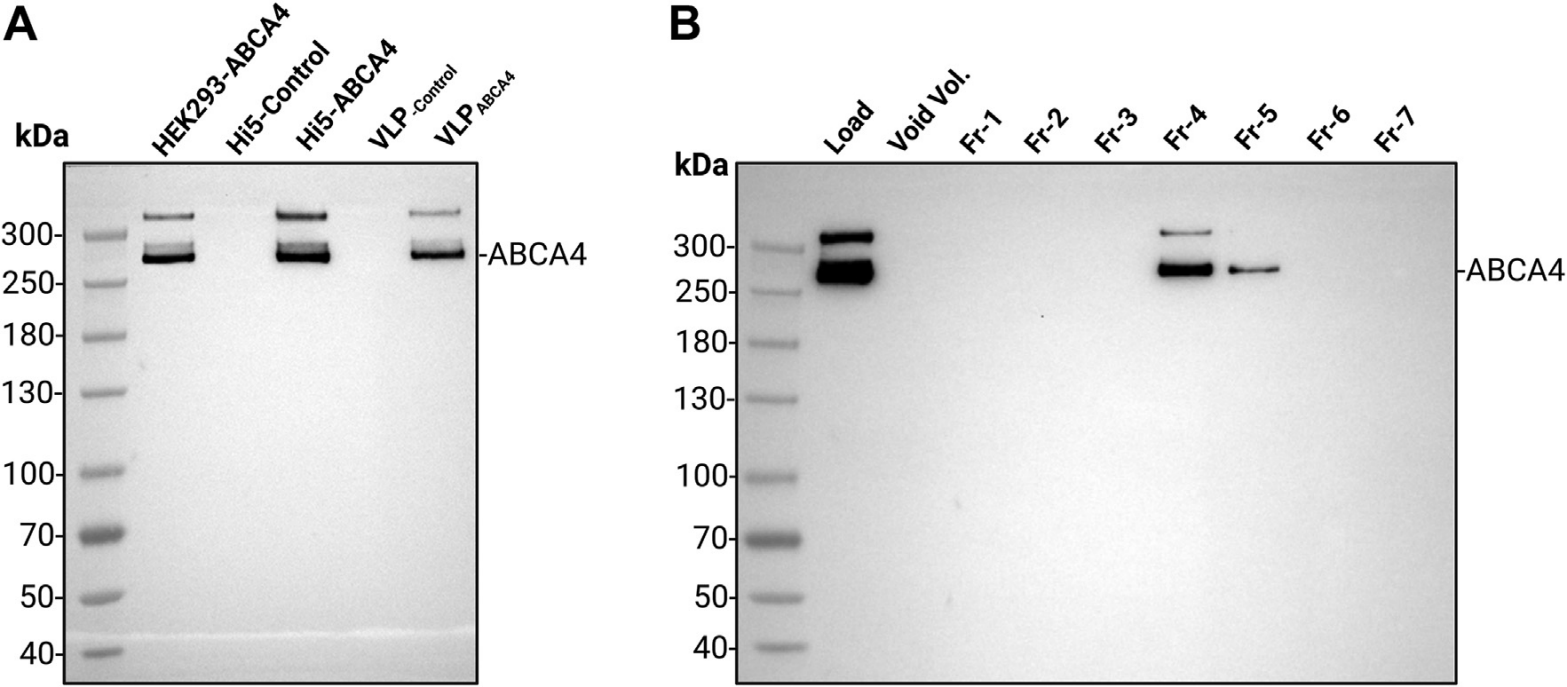
**ABCA4 protein expression and VLP-targeting analysis.***A*, Western blot of ABCA4 protein expression in High Five (Hi5) insect cell lysates and VLP preparations using antibody against ABCA4. From *left to right*, lanes represent: Spectra Multicolor High Range Protein Ladder (Cat# 26625), ABCA4 from HEK293 lysate (positive control), Hi5 cell lysate infected with VLP-producing negative control baculovirus (negative control), ABCA4-VLP–producing Hi5 cell lysate, control VLP, and ABCA4-VLP. Two distinct bands were observed, with the top band likely indicative of posttranslational modifications occurring in ABCA4. Only the ABCA4-expressing cells and ABCA4-VLP samples show a specific ABCA4 signal. Samples were loaded based on an equal total protein concentration, as determined by the Bradford assay. *B*, Western blot analysis of the purified ABCA4-VLP fractions with size-exclusion chromatography (Izon qEV1/70 nm). The initial 2.8 ml was pooled as the default buffer volume (void volume). Fractions were loaded as 20 μl. VLP, virus-like particle.

The Bac-to-Bac Baculovirus Expression System was employed to achieve transient expression of ABCA4-VLPs in insect cell lines. The expression of ABCA4 in High Five insect cells was confirmed through Western blot analysis (WBA) of cell lysates, using an ABCA4 domain–specific antibody as a probe. The antibody detected a clear band corresponding to the expected molecular weight of ABCA4 and comparable to that of the control ABCA4 protein produced in the HEK 293 cell line (Fig. 3*A*). An additional higher molecular weight band was observed, likely indicative of posttranslational modifications occurring in ABCA4 (62, 63) (Fig. 3*A*). To confirm the presence of ABCA4 in the VLPs, in parallel, WBA of ABCA4-VLPs was carried out. The results identified comparable-sized ABCA4 bands, suggesting successful protein folding and targeting of the protein to the cell surface plasma membrane, leading to its incorporation of the protein into self-assembling VLPs (Fig. 3*A*). The purified ABCA4-VLP samples showed high recovery, as confirmed by the WBA after size-exclusion column purification (Fig. 3*B*).

### Characterization of ABCA4-VLPs

#### ABCA4 expressed on insect cell plasma membrane maintains a uniform and native topology

**Figure 4:**
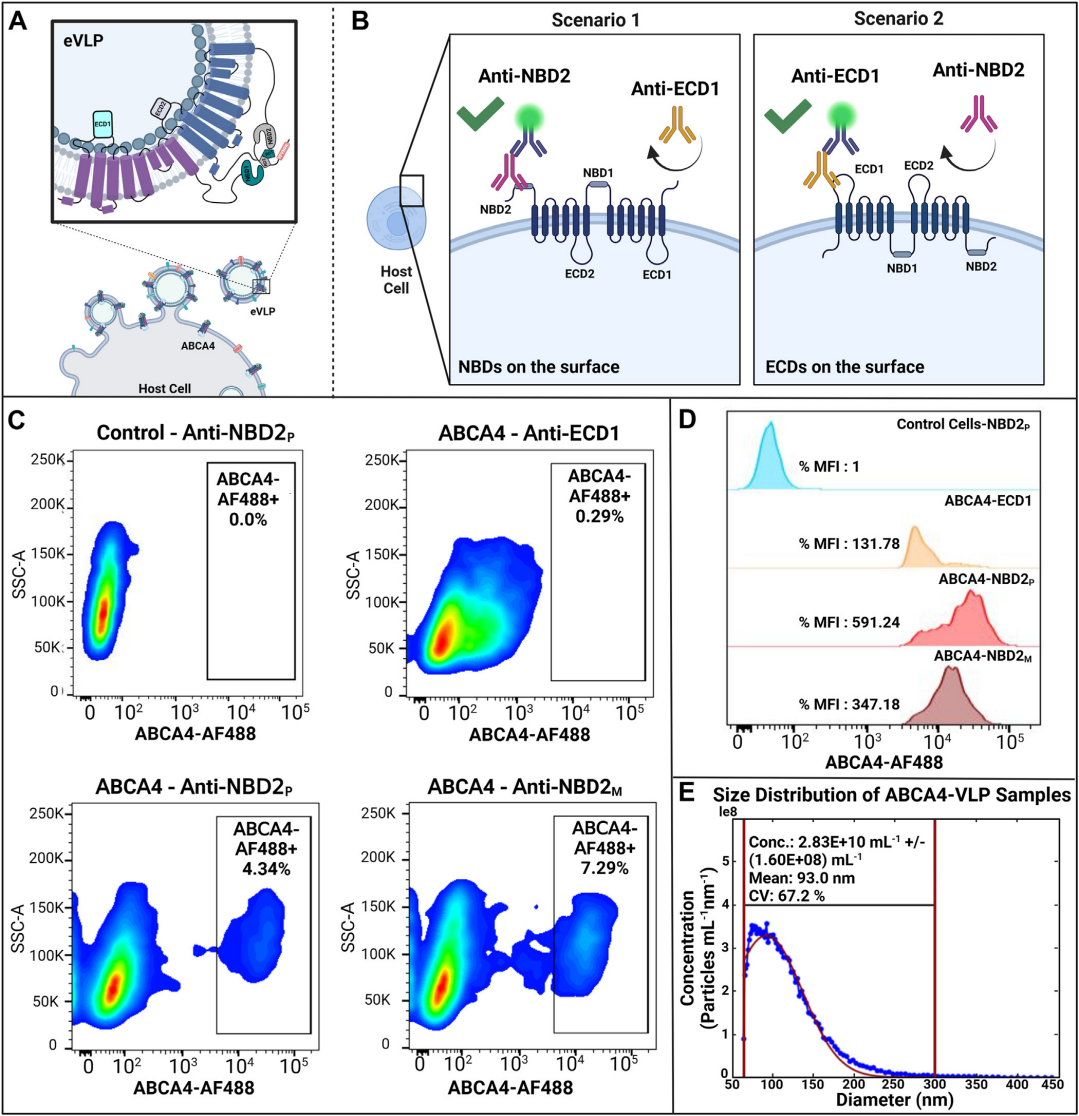
**ABCA4 is expressed with NBD domains projecting outwards from the lipid bilayer of the VLP.***A*, illustration demonstrating the principle that the VLP surface topology mirrors the host cell surface. This similarity arises because the VLP buds off unidirectionally from the host cell, thus inheriting its membrane characteristics. *B*, flow cytometry experimental design to assess membrane topology of the ABCA4 protein expressed in insect cells. *C*, representative flow cytometry analysis of Sf9 cells using domain-specific antibodies demonstrated a uniform topology of the ABCA4 in the cell plasma membrane, with NBDs outward facing, supporting a native and uniform topology of ABCA4 in the VLP assembly. The representative plots depict the following: VLP-expressing cells without ABCA4 stained with an NBD2-specific polyclonal antibody (*top left*), ABCA4-VLP–expressing cells stained with an ECD1-specific antibody (polyclonal) (*top right*), and ABCA4-VLP–expressing cells stained with NBD2-specific polyclonal and mAbs (*bottom left and right*, respectively). *D*, histograms showing the median fluorescence intensity (MFI). *E*, ABCA4 is produced in VLPs of uniform size distribution. Microfluidic resistive pulse sensing (MRPS) analysis (Spectradyne nCS1) was used to determine the size distributions of ABCA4-VLPs in solution. The diluted VLPs were evaluated in triplicate experiments at a temperature of 23 °C, and the results were combined to generate the illustrated size distribution of ∼93 nm (created with BioRender.com). ECD1, extracytoplasmic domain 1; NBD, nucleotide-binding domain; VLP, virus-like particle.

The native topological arrangement of ABCA4 in the photoreceptor outer segment discs is characterized by its nucleotide-binding domians (NBDs) facing the cytoplasm and extracytoplasmic domains (ECDs) oriented toward the disc lumen, and this is essential for its proper functioning *in vivo*. To investigate the topological orientation of the ABCA4 protein within the lipid-bilayer structure in our *in vitro* system, we employed flow cytometry analysis to assess the cell surface expression of ABCA4 in the insect cells. Given that the transmembrane protein ABCA4 is incorporated into the enveloped VLPs through budding from the cell plasma membrane, we inferred that the topological organization of ABCA4 on the cell surface would reflect that of the VLP structure (Fig. 4*A*).

To examine ABCA4 protein orientation in the membrane, we utilized topologically opposite ABCA4 antibodies, two targeting the NBD2 domain and another specific to an epitope on the ECD1 domain, followed by an indirect detection using a secondary antibody conjugated with the AF488 fluorophore (Fig. 4*B*). We observed a strong AF488 fluorescence signal with the NBD2-specific antibodies relative to that observed with the ECD1-specific antibody. The weak signal observed with ECD1 antibody staining was likely attributable to nonspecific binding to the cell surface (Fig. 4, *C* and *D*). The negative control experiments, including isotype controls, secondary antibody-only, and empty VLP staining, did not give any signal (Fig. S1). Our findings confirm that ABCA4 maintains its native topology in the VLP assembly, with NBDs oriented toward the external side of the insect cell plasma membrane, akin to their cytoplasmic orientation in the outer segment discs of photoreceptor cells, while the ECDs remain inside the cells, corresponding to their luminal localization (Fig. 4, *A*–*D*).

#### Nanoparticle size analysis

To evaluate the size distribution, homogeneity, and concentration of the purified VLP preparation, nanoparticle size analysis was performed using microfluidic resistive pulse sensing (Spectradyne nCS1). We observed a single peak with a mean diameter of 93 nm for the particles (Fig. 4*E*). This analysis also indicated a high yield of particles in the culture media with a concentration of 5.66 × 10^10^ ml^-1^ ± 3.20 × 10^8^ ml^−1^ post-qEV column purification, further supporting efficient production and release of VLPs from the insect cells (Fig. 4*E*).

### ABCA4 produced in VLPs demonstrates retinal stimulated ATPase

#### Basal and retinal stimulated ATPase of the WT ABCA4 in the VLPs

**Figure 5:**
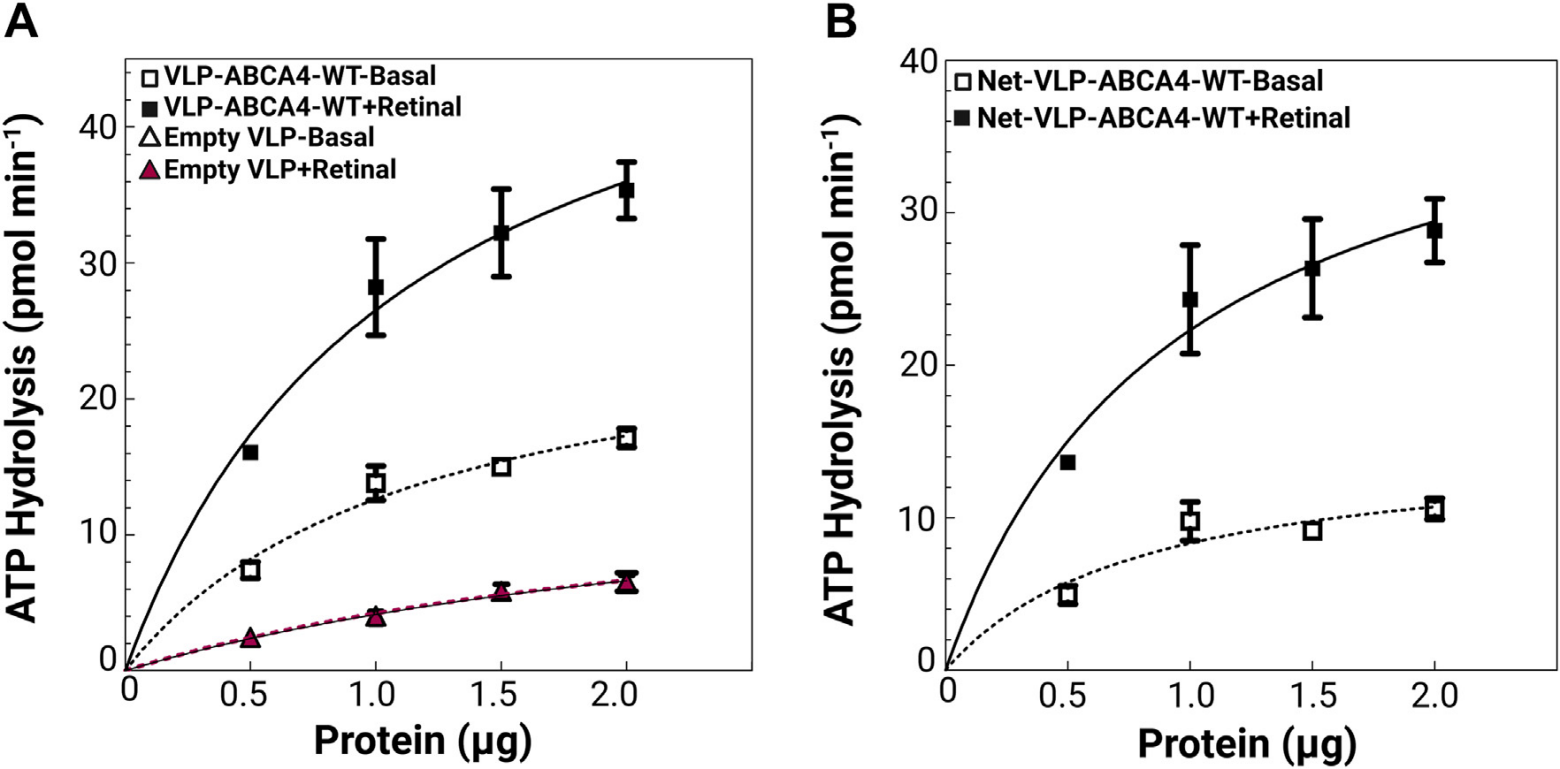
**ABCA4-VLPs maintain functional activity and specifically demonstrate retinal-stimulated ATPase.***A*, ATPase activity of empty VLPs and ABCA4-VLPs were measured in the presence or absence of 40 μM all-*trans*-retinal in the presence of PE (DOPE). All VLP samples were diluted to 1 μg/μl total protein concentration using the Bradford assay, and ATP hydrolysis rates are presented as pmol/min.μg of total VLP protein concentration. The reactions were incubated at 37 °C for 60 min under low light conditions. *B*, blank subtracted analysis illustrating the ABCA4 attributable ATP hydrolysis in the VLPs. The error bars represent the SD, based on assays performed in independent biological triplicate. DOPE, 1,2-dioleoyl-sn-glycero-3-phosphoethanolamine; VLP, virus-like particle.

To verify the functional integrity of ABCA4 within VLPs, we assessed the basal and retinal stimulated ATPase activity of ABCA4 in the VLPs. Assaying retinal stimulated ATPase was chosen as it is a good readout of ABCA4 functionality as it simultaneously examines both nucleotide hydrolysis and substrate (retinal) interaction, specific for ABCA4. ATP hydrolysis was measured under basal conditions and with retinal stimulation (in the presence of all-*trans*-retinal [ATR]-1,2-dioleoyl-sn-glycero-3-phosphoethanolamine [DOPE] mixture forming N-retinylidene-phosphatidyl ethanolamine (NRPE)) using an α-^32^P-ATP TLC-based assay (64, 65, 66). To differentiate the nucleotide hydrolysis activity associated specifically with ABCA4 and exclude the influence of other potential membrane proteins native to High Five insect cells, we subtracted the ATPase activity of the negative control VLPs from that of the ABCA4-VLP. We found that ABCA4, expressed in the VLPs, was able to hydrolyze ATP in the rate of 7.78 pmol/min.μg of total VLP protein concentration, and this rate is increased to 20.9 pmol/min.μg in the presence of retinal substrate (Fig. 5, *A* and *B*). Our findings indicate that ABCA4 expressed in the VLPs retains its enzymatic activity.

### ABCA4 produced in VLPs as a useful platform for the analysis of retinal degenerative disease-associated genetic variants

To determine the efficacy of the VLP platform for studying ABCA4 variants, we examined two known Stargardt disease–associated pathogenic ABCA4 variants, p.N965S and p.C1488R (23, 53, 67, 68, 69, 70), in the VLP platform.

#### ABCA4 variant p.N965S is found at substantially reduced levels in VLPs

**Figure 6:**
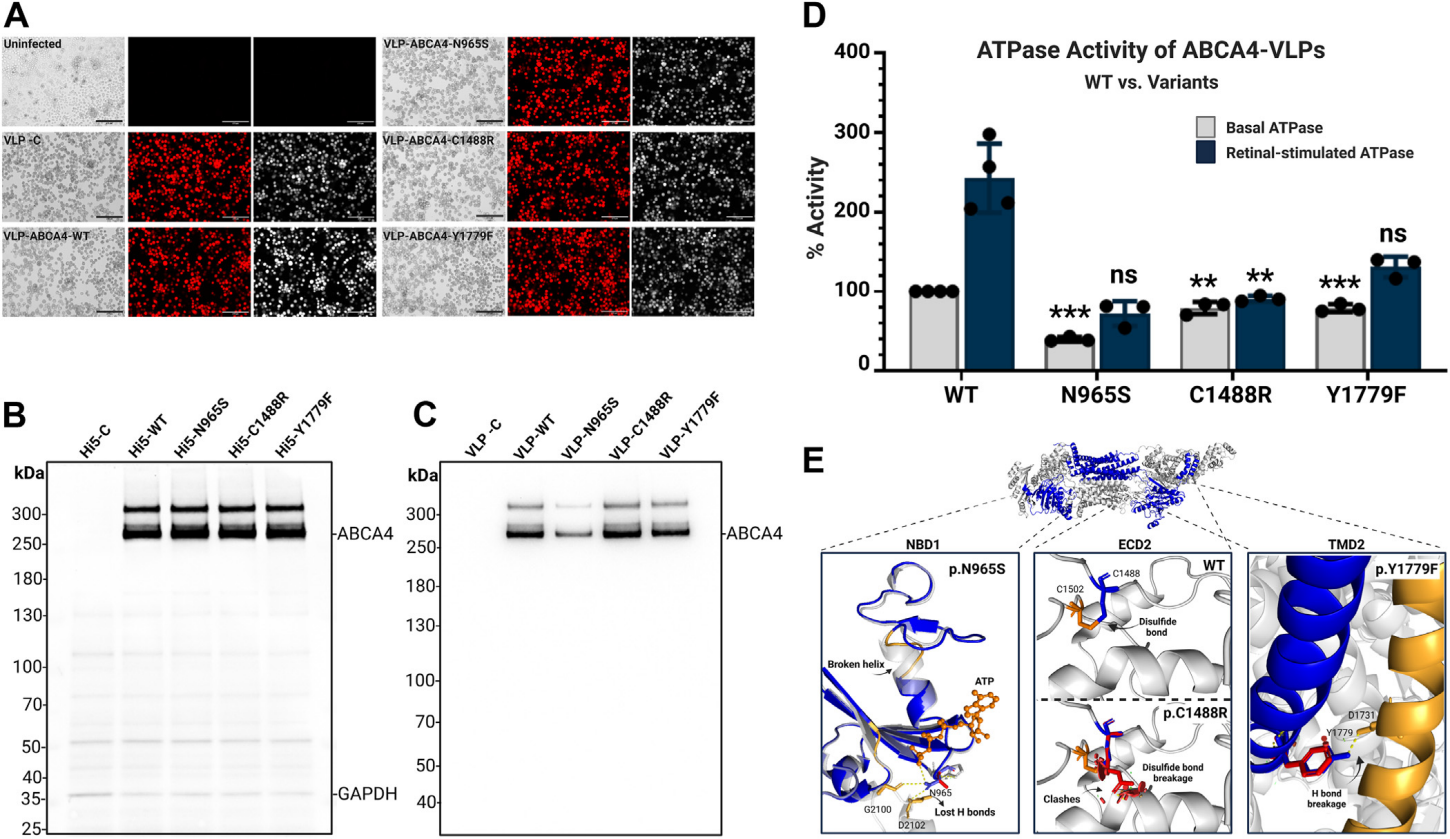
**Comparative analysis of WT and variant ABCA4 proteins.***A*, fluorescence microscopy images of Hi5 cells depicting the mCherry reporter expression, indicating successful baculovirus infection. The infection rates are compared across cells expressing empty VLPs (-control), VLP-ABCA4 WT and variant proteins. Scale bars represent 275 μm. *B*, Western blot analysis showing the expression levels of ABCA4 in High Five (Hi5) insect cell lysates for WT and variants N965S, C1488R, and Y1779F, with the expression of GAPDH as a loading control. Samples were loaded based on 5 μg total protein concentration, as determined by the Bradford assay. *C*, Western blot analysis of ABCA4 levels in virus-like particle (VLP) samples, with 10 μl of each sample loaded. Quantitative analysis of independent triplicate blots is provided in Table S2. *D*, graph comparing the basal and retinal-stimulated ATPase activity of ABCA4-VLPs for WT and each variant. The data represents the mean of relative enzymatic activity ± SD for n ≥ 3, where each point represents independent biological replicate experiment. Statistical analysis of basal activities was performed using one-way ANOVA followed by Tukey post hoc comparisons. ANOVA explained 97.1% of the variation (F(3,9) = 99.8, *p* < 0.0001). Post hoc tests showed significant differences between WT and N965S (*p* < 0.0001), Y1779F (*p* = 0.0009), and C1488R (*p* = 0.001). For retinal-stimulated activity (fold change), ANOVA explained 71.1% of the variation (F(3,9) = 7.4, *p* = 0.0084), with a significant difference between WT and C1488R (*p* = 0.0056). ∗∗*p* < 0.01 ∗∗∗*p* < 0.001 *versus* WT. ∗∗*p* < 0.01 ∗∗∗*p* < 0.001 *versus* WT. *E*, *in silico* protein structure analysis, pinpointing the localization of variants within the context of the overall three-dimensional structure of ABCA4. As previously shown (54), the Asn-965 residue is located in the Walker A sequence of NBD1 and binds to the ATP molecule, which is disrupted with the Ser substitution. In contrast, the p.C1488R variant is in the ECD2, which has been shown to be involved in the substrate interaction. The substitution at Y1779F leads to a loss of a hydrogen bond, which is likely critical for transmembrane domain 2 (TMD2) intradomain interaction (created with BioRender.com). ECD2, extracytoplasmic domain 2; NBD, nucleotide-binding domain.

We investigated the potential impacts of variants on protein folding and membrane localization by comparing the ABCA4 targeting levels to VLPs. We first ensured uniform infection rates across samples, confirmed by consistent mCherry reporter gene expression (Fig. 6*A*), and equivalent ABCA4 expression levels in Hi5 cell lysates across WT and variants (Fig. 6*B*), which allowed us to attribute any observed differences in ABCA4 membrane targeting or activity directly to the effects of the variants. Furthermore, we ensured uniformity of purified VLP particles by confirming that the SDS-PAGE profiles of the VLPs demonstrated comparable levels of endogenous membrane proteins, indicating that different levels of ABCA4 in the VLP samples are not due to variations in the sample preparation.

Despite similar expression levels in cell lysates, WBA of purified VLP samples revealed a significant reduction in ABCA4 levels for the p.N965S variant with only 49% of WT levels (Fig. 6*C* & Table S2). This suggests that the p.N965S variant leads to protein misfolding, preventing its proper localization to the membrane.

#### Functional characterization of the ABCA4 disease–associated variants p.N965S and p.C1488R

To assess the impact of the N965S and C1488R variants on the enzyme function of ABCA4, we compared their basal and retinal-stimulated ATPase activity with WT ABCA4-VLPs. Titration experiment of VLP-ABCA4-WT was performed to determine the optimal enzyme concentration, where maximum hydrolysis occurs while the activity remains within the linear range (Figs. S2 and S3). Based on this assessment, we chose to use 1 μg of each VLP sample for the comparison. At this concentration, the ATPase activity of the WT exhibited a 2.2-fold stimulation in the presence of the retinal substrate (NRPE).

The basal ATPase activity of the N965S variant was only 39.8% of that observed in WT ABCA4 (*p* < 0.001), highlighting a substantial decrease, which could be reflective of the decreased targeting of the variant protein to the VLPs. In contrast, the C1488R variant retained its activity at approximately 80% of the WT level (*p* < 0.01) (Fig. 6*D*). The N965S ABCA4 basal level ATPase activity was stimulated by 1.8-fold in the presence of NRPE, indicating a substantial, though attenuated response compared to WT, which is not statistically significant difference. (Fig. 6*D*). In contrast, for the C1488R ABCA4 variant, no appreciable stimulation of ATPase activity was observed in the presence of NRPE (*p* = 0.0056), highlighting a distinct response profile between the two variants.

### Functional characterization and pathogenicity assessment of a VUS, p.Y1779F

In parallel with our analyses of the known pathogenic variants, we extended our investigation to include a VUS, p.Y1779F, as a proof of concept of the utility of this platform to assess VUS. VLPs harboring ABCA4 p.Y1779F were produced and purified. Its expression was analyzed by WBA and ATPase activities were determined.

#### p.Y1779F maintains membrane targeting

Consistent with our approach for the previously characterized variants, we ensured uniform infection rate and expression level compared to WT, as evidenced by mCherry reporter, and WBA of cell lysates (Fig. 6, *A* and *B*). The WBA of VLPs indicated that the Y1779F variant was successfully targeted to and incorporated into the VLP membrane at levels equivalent to the WT protein, implying correct membrane targeting of this variant (Fig. 6*C*). Furthermore, flow cytometry analysis revealed that the topological orientation of the p.Y1779F variant remained unchanged, exhibiting the same topology as the WT ABCA4 in the cell plasma membrane (Fig. S4).

#### p.Y1779F variant exhibits altered ATPase activity

p.Y1779F variant demonstrated 79% of the WT basal ATPase activity (*p* = 0.0009). However, its ATPase activity showed approximately 1.7-fold retinal stimulation, compared to the 2.2-fold increase observed in the WT, indicating reduction in the variant's response to its substrate, which is statistically not significant (Fig. 6*D*).

#### p.Y1779F variant may affect protein structure

Computational analysis of the ABCA4 structure revealed that the Tyr-1779 residue plays a crucial role in maintaining intradomain interactions, particularly by forming a hydrogen bond with a residue located in an adjacent α-helix. As shown in Figure 6*E*, the substitution of Tyr-1779 with phenylalanine disrupts this interaction, which may be the reason behind the protein’s functional impairment.

Overall, the p.Y1779F variant demonstrates structural and functional alterations as evidenced by both *in silico* structure predictions and *in vitro* functional characterization. This variant's absence in population databases, its occurrence at a highly conserved residue, and its classification as likely pathogenic by multiple pathogenicity prediction tools; PolyPhen-2 (71), REVEL (72), CADD (73), and MutationTester (74) suggests a probable pathogenic impact.

## Discussion

We sought to develop a simple, high-throughput approach to assess the consequences of *ABCA4* genetic variants on protein function without the need for detergent solubilization, purification, and reconstitution. This method would be particularly valuable for the analysis of ABCA4 VUS as well as other clinically important membrane proteins. Our results demonstrated the feasibility of the VLP platform for the expression and functional characterization of the ABCA4 transmembrane protein and its variants. Our findings showed that the ABCA4 protein expressed in insect cells maintains a native and uniform topology, and it is biologically active with respect to retinal-stimulated ATPase activity within the VLP structure. Additionally, we demonstrated the platform's efficacy through the *in vitro* characterization of two clinically relevant and well-studied pathogenic ABCA4 genetic variants, namely p.N965S and p.C1488R. These variants have been identified in numerous individuals affected by the disease, including some cases of homozygosity (23, 53, 67, 69, 75, 76, 77, 78, 79, 80).

Previously, the capture of multipass membrane cell surface proteins in VLPs has been successfully employed in mammalian cell cultures, including G protein–coupled and insulin receptors, for the study of ligand–protein interactions as immunogens and therapeutic agents (81, 82, 83, 84). In this study, we utilized an insect cell expression system to produce the VLPs. We have been able to successfully express ABCA4 in insect cells along with concomitant, robust trafficking to the VLPs; to the best of our knowledge, this represents the first report of an ABC transporter in insect cell–derived VLPs, and also the first study that employs the VLP system as a platform for the functional characterization of a membrane protein and its disease-associated variants.

In our insect cell–based expression system, we observed that the WT ABCA4 protein was effectively directed to the plasma membrane. This was evident by its presence in isolated VLPs, which, due to their formation and secretion processes, selectively carry cell membrane surface proteins. Flow cytometry analysis further confirmed the efficient targeting of ABCA4 to the insect cell surface in our system. This contrasts with some earlier studies that transiently expressed ABCA4 in mammalian cell cultures, where it was found that even the WT ABCA4 was primarily located in internal membranes (48, 55, 56, 70, 85). Nevertheless, a recent study reported that WT ABCA4 is expressed on the plasma membrane in HEK-293T mammalian cells (86). Furthermore, the rhodopsin protein, which shares the native location with ABCA4, has also been shown to localize on the surface of mammalian cells *in vitro* (87, 88, 89). The reasons behind this variable localization of ABCA4 in different heterologous expression systems are not obvious.

Pathogenic *ABCA4* missense variants can be those that impact protein structure and function, leading to loss of ABCA4 enzymatic function; although in some cases, an amino acid substitution is associated with protein misfolding, resulting in retention in the Golgi/endoplasmic reticulum and a failure to target the outer segment discs of the photoreceptors. The phenotypic impact of a misfolding variant can be highly significant since the protein never reaches its required cellular destination (87). In our study, we were able to easily identify the N965S variant in the VLP platform as a variant that led to misfolding. Although we found that the N965S was expressed at the same levels as its WT counterpart, its limited ability to integrate in the VLP suggested failure to be targeted to the membrane, consistent with previous reports on this variant conducted in a knockin mouse model showing its retention in the Golgi/ER (68). Comparison of the ABCA4 levels in VLPs of the C1488R variant with WT revealed no differences, suggesting that the pathogenicity of this variant is not due to Golgi/ER retention. Although several reports support the pathogenicity of the C1488R (7, 53, 67), we were unable to locate any *in vitro* or *in vivo* studies that describe the cellular mistargeting of this variant, supporting that the deficit arises from the loss of protein function.

The ATP-driven transport of retinoids in the photoreceptor outer segment discs is a well-known biological function of ABCA4, which is essential for the continued operation of the visual cycle (9, 46). Numerous research groups have established that the ABCA4 protein ATPase activity is stimulated by all-*trans* retinal, as well as its Schiff’s base NRPE and its derivatives (55, 56). Our study confirmed that the WT ABCA4 protein expressed using the VLP platform exhibits a 2.2-fold retinal stimulation in ATPase activity, similar to what is observed in other expression systems. For instance, ABCA4 expressed in HEK293 cells and reconstituted into proteoliposomes exhibits a retinal-stimulated increase of ≥2-fold (70, 85, 90, 91). This comparison highlights the consistency of our VLP-based system with established expression systems. Using this platform, we have examined two well-characterized ABCA4 variants, N965S and C1488R, to validate the efficacy of the VLP system in assessing the effects of missense variants.

The N965S variant is one of the most common ABCA4 variants in Danish and Chinese populations (76, 77, 78). Our data indicate that the reduced plasma membrane representation of the N965S variant is only 49% of WT levels and likely accounts for the decrease in ATPase activity observed relative to the WT. The evaluation of variant protein activity using the VLP platform revealed that ABCA4 p.N965S retained its retinal-stimulation of basal level ATPase activity. In contrast to our observations, a previous study analyzing the p.N965S variant reported a complete loss of retinal-stimulated ATPase (68). However, this investigation analyzed recombinant ABCA4 protein that was extracted, purified, and reconstituted from mammalian cell lines, which may account for the observed differences. Quazi *et al.* reported, this variant has exhibited 37% of the NRPE transfer activity relative to the WT ABCA4 (92). The p.N965S variant localizes to the Walker A nucleotide binding motif of the NBD1, which interacts with the ATP molecule (Fig. 6*E*) and, therefore, would be expected to impact the ATPase activity of this domain (51). Despite this, the consistent rate of basal activity with VLP targeting and the maintained retinal stimulation of ATPase activity in the N965S variant indicate that the protein retains functional capacity within the VLP system. This retention is likely due to the complex interaction network within the protein, where multiple residues, including those in NBD2, contribute to ATP binding and hydrolysis. This nuanced understanding highlights the importance of considering both membrane-targeting levels and intrinsic enzymatic activity when assessing the impact of genetic variants. These consequences are consistent with clinical reports that this mutation exerts a significant and early impact on retina function (76, 77, 78).

Consistent with previous reports examining the biochemical consequences of the p.C1488R, this variant produced in the VLP platform displayed a minor attenuation of basal ATPase activity while losing almost all retinal stimulation. These results are also in line with our previous findings indicating reduced *ATR* binding affinity of this variant protein (93). This variant has been previously shown to have a destabilizing effect on the protein structure *in silico*, with the disruption of a disulfide bond between residues Cys-1488 and Cys-1502 (51), which, based on the cryoEM structure of the protein, is important in maintaining the retinal-binding pocket (Fig. 6*E*) (51, 90, 94), helping to explain our findings.

Our VLP-based *in vitro* functional assessment reveals that the Y1779F variant exerts a moderate, yet discernible, deleterious impact on ABCA4 protein function. Categorized as a VUS in the ClinVar database, the Y1779F variant exhibits a diminished response to retinal stimulation. Examination of this variant by flow cytometry demonstrated that the topological orientation of the p.Y1779F variant remained unchanged, exhibiting the same topology as the WT ABCA4 in the cell plasma membrane. *In silico* structural analyses further highlight the molecular implications of these variant, demonstrating disrupted intradomain interactions within ABCA4. Such molecular alterations are likely contributors to the functional deficits we observed in our experimental assays. While these findings suggest a potential pathogenic impact, further studies, including transport assays, are necessary to definitively classify the pathogenicity of Y1779F.

The VLP-based expression system appeared to be a valuable tool for the expression of large transmembrane proteins like ABCA4 by simplifying the expression and purification process while preserving the protein's native-like environment for functional studies and pathogenicity analysis. However, additional assays would be needed to detect and distinguish other variants that uniquely affect the transport event itself. With that said, the fundamental attributes that would be necessary for transport, such as retinal-stimulated ATPase, can be assessed in the VLP platform. Findings from the VLP characterization platform aligned well with and extended the existing literature on *ABCA4* variants, demonstrating the efficacy of this method in the functional analysis of genetic mutations associated with retinal diseases (Table 1). Our study supports the application of the VLP platform for future research on *ABCA4*-related inherited retinal degenerations, providing valuable insights into disease mechanisms and facilitating pathogenicity prediction of the large number of *ABCA4* genetic variants of unknown clinical significance.

**Table 1:** Comparative analysis of functional impacts on ABCA4 variants: Previous Findings *versus* current study

Variant	Previous findings	This study
c.2894A>G:p.N965S	• Misfolding and Golgi retention in a knockin mouse model (68). • Decreased basal and retinal-stimulated ATPase activity (68). • Reduced the N-retinylidene-PE transfer activity (92).	Suggests misfolding and retention in Golgi/ER based on reduced VLP targeting. Reduced basal ATPase activity but retinal stimulation is largely retained.
c.4462T>C:p.C1488R	Disrupted a disulfide bond (91), reduced retinal binding affinity (93) Decreased ATPase activity in the presence of titrated ATR (70).	Minor decrease in basal ATPase activity and significant loss of retinal stimulation.
c.5336A>T:p.Y1779F	N/A (VUS)	• *In silico* analysis indicates disruption of intradomain interactions. • Minor reduction in basal ATPase activity and diminished response to retinal stimulation.

Abbreviations: VLP, virus-like particle; VUS, variants of unknown significance.

This table summarizes the functional characterization of ABCA4 variants c.2894A>G:p.N965S, c.4462T>C:p.C1488R, and c.5336A>T:p.Y1779F and compare our VLP-based characterization findings with previous findings.

*In vitro* functional studies of *ABCA4* variants are crucial for the enhanced evaluation of which sequence variants are pathogenic and which are benign population SNPs. Nevertheless, traditional methods for purifying and characterizing transmembrane proteins, like ABCA4, face various limitations. In this study, we successfully utilized a VLP platform to express and purify functionally active ABCA4 transmembrane protein in an insect cell culture system. This approach offers significant advantages in terms of simplicity and efficiency, enabling the higher throughput functional characterization of ABCA4 protein variants. This approach could aid in improving diagnostic precision by reclassifying the disease-causing *ABCA4* variants of uncertain significance and, quite plausibly, broader applicability to other transmembrane proteins and their genetic-related disorders.

## Experimental procedures

### Reagents and buffers

ATP-α-32P was purchased from PerkinElmer; ATR from Sigma-Aldrich (Cat. #R2500), DOPE (18:1 (Δ9-Cis) phosphatidylethanolamine (PE)) from Avanti Polar Lipids (Cat. # 850725C), Halt protease inhibitor from Thermo Fisher Scientific (Cat. # 78425) and TLC plates from Sigma-Aldrich (Cat. #Z122882). The following buffers were used in the study: Dulbecco's phosphate-buffered saline (DPBS) (Cat. #14040133, Thermo Fisher Scientific): 1X, filtered through 0.22 μm filter; tris-buffered saline containing 0.1% Tween 20 (TBS-T): 10 mM Tris–HCl, pH:8, 150 mM NaCl, 0.1% (v/v) Tween-20; radioimmunoprecipitation assay buffer: 50 mM Tris–HCl, 150 mM NaCl, 1.0% (v/v) NP-40, 0.5% (w/v) sodium deoxycholate, 1.0 mM EDTA, 0.1% (w/v) SDS, and 0.01% (w/v) sodium azide, pH 7.4; buffer A: 2% fetal bovine serum in 1X DPBS filtered through 0.22 μm filter; buffer B: 0.1% bovine serum albumin (w/v) in DPBS clarified with an Amicon Ultra-4100-kDa centrifugation filter; buffer C: 25 mM Tris–HCl, pH 7.5, 10% glycerol, 0.1 mg/ml bovine serum albumin, 5 mM MgCl_2_, and 5 mM DTT.

### Cell culture

*Spodoptera frugiperda* 9 (Sf9) (Cat. # 12659017, Thermo Fisher Scientific) and Trichoplusia ni BTI-TN5B1-4 (High Five, Hi5) (Cat. # ENH127-FP, Kerafast) cells were cultured following the vendor's recommended protocols. The Sf9 cells were cultured in Gibco SF900-III serum-free medium (Thermo Fisher Scientific) without the addition of antibiotics or supplements, while Hi5 cells were supplemented with 5 mM L-Glutamine.

### Expression and purification of ABCA4-VLPs in BEVS

ABCA4-VLP expression in BEVS was conducted as described in the Bac-to-Bac Baculovirus Expression System instruction manual (Invitrogen Life Technologies) with the following modifications. The native *ABCA4* coding sequence corresponding to amino acids 1 to 2273 of the full-length ABCA4 protein based on the genome assembly GRCh38:Chr1:83457325-104273917, and Reference Transcript NM_000350.3 was cloned into the pFastBac Dual (pFBD) donor plasmid. The pFBD-ABCA4 construct was transformed into competent DH10VLPFactory cells along with empty pFBD to serve as a negative control in downstream experiments.

To generate VLP-producing baculovirus, transfection of the insect cells with bacmid construct was performed. Sf9 cells, at a density of 1 × 10^6^ cells/ml, were transfected with the bacmid construct containing the *ABCA4* coding sequence. A negative control construct was used to generate control VLPs, lacking the ABCA4 gene, for comparative purposes. Transfections were carried out using a 1:1 DNA-to-reagent ratio in the 6-well plate setting. Following transfection, cells were maintained at 27 °C for 72 h to facilitate baculovirus production. The resulting initial titer (V_0_) was used to achieve a higher titer V_1_ virus by infecting Sf9 insect cells at a 40 to 60% confluency with 0.5 ml of V_0_ in the T75 flask setup. The V_1_ baculovirus stock was harvested with the cell culture medium 72 h posttransfection and used to infect the insect cells for VLP expression.

The High Five cell line was chosen for ABCA4-VLP expression due to its superior protein expression capacity. Adherent High Five cells, cultured in a T182 flask and supplemented with 5 mM L-Glutamine, were infected with 0.40 ml of the V_1_ stock. This was followed by a 2-h incubation at 27 °C with shaking at 60 rpm in the dark, and then a 72-h static incubation. After this period, the culture media, containing VLPs, was harvested for purification.

Throughout the process, the efficiency of baculovirus infection was monitored by observing mCherry reporter gene expression under an inverted fluorescence microscope after each transfection and infection. Additionally, cells were lysed using radioimmunoprecipitation assay buffer, and the resulting cleared lysates were subjected to WBA to confirm ABCA4 expression.

### *In vitro* site-directed mutagenesis of the pFBD-ABCA4 construct

To create the variant constructs, c.2894A>G:p.Asn965Ser, c.4462T>C:p.Cys1488Arg, and c.5336A>T:p.Tyr1779Phe, site-directed mutagenesis was carried out using the Quik-Change Lightning Site-Directed Mutagenesis Kit (Agilent) following the manufacturer's protocol. The primers used for mutagenesis are provided in Table S1. Prior to generating the recombinant ABCA4-bacmid construct for the variants, the desired mutations and absence of unintended mutations were confirmed by DNA sequencing.

### Isolation and purification of the VLPs

ABCA4-VLPs were recovered from the cell culture media through the following steps. The media was clarified through centrifugation at 500*g* for 10 min to remove floating cells, followed by centrifugation at 3000*g* for 30 min at 4 °C to eliminate unwanted cellular debris and bigger particles. Subsequently, VLPs were isolated through sedimentation at 100,000*g* at 4 °C for 4 h. The resulting VLP pellet was resuspended in DPBS.

For further purification of the VLP sample, Izon qEV1/70 nm columns (IZON Science) were used according to the manufacturer's instructions, and 0.7 ml fractions were manually collected with DPBS. The fractions confirmed to contain ABCA4-VLPs *via* WBA were then supplemented with 2 mM of DTT and 1X protease inhibitor.

### Western blot analysis

WBA was conducted to confirm the expression of ABCA4 in the insect cells, the presence of ABCA4 in the purified VLP samples, and to evaluate the impact of ABCA4 variants on protein folding and membrane localization, by comparing the ABCA4 levels in purified VLPs across variants after establishing a uniform baseline, achieved by a consistent mCherry reporter signal and uniform cell-level expression of ABCA4 across all samples. WBA was carried out based on the protein concentration determined by the Bradford assay (95). An ABCA4-specific antibody targeting the nucleotide-binding domain 2 (NBD2) (anti-NBD2-Rabbit;Cat. # ab72955, Abcam) was used as a probe. Proteins were separated by SDS-PAGE using a 3 to 8% precast tris-acetate gel (Cat. # EA0378BOX, Invitrogen Life Technologies). Following transfer to the nitrocellulose membrane (Cat. #1704158, Bio-Rad), the membrane was blocked in 5% nonfat dry milk in TBS-T for 60 min. Primary antibody (anti-NBD2-Rabbit, diluted 1:1000 in 1% nonfat dry milk in TBS-T) was added and incubated for 60 min at room temperature, followed by three 10-min washes in TBS-T. The membrane was then incubated with anti-rabbit horseradish peroxidase secondary antibody (Abcam, ab205718) (diluted 1:10,000 in 1% nonfat dry milk in TBS-T) for 30 min and washed with TBS-T. In addition, a GAPDH loading control mAb (Cat. #MA5-15738-horseradish peroxidase) was used for the comparison of cell-level protein expression across WT and variants. For this, after secondary antibody staining and additional three washing steps of 10 min each, the membrane was incubated with anti-GAPDH (diluted 1:1000 in 1% nonfat dry milk in TBS-T) for 1 h and then washed three times in TBS-T. Membranes were subjected to chemiluminescence detection using Radiance Plus Chemiluminescent Substrate, Azure Biosystems (Cat# AC2103), then visualized, and analyzed using an iBright imaging system (Thermo Fisher Scientific).

### Flow cytometry analysis

To determine the topological organization of the ABCA4 transmembrane protein in this expression system, flow cytometry analysis was performed. ABCA4-expressing cells were analyzed using specific antibodies targeting different domains of ABCA4 to identify the orientation of either ECDs or NBDs toward the outer membrane. Empty VLP-expressing cells and uninfected cells were used as negative controls along with the appropriate isotype controls. Briefly, 48 h postinfection, 1 × 10^6^ Sf9 cells were washed three times with buffer A and stained with 10 μg/ml of either ECD1-specific primary antibody (Cat. # A10556, ABclonal), NBD2-specific primary antibody (3F4-mouse monoclonal and anti-NBD2-Rabbit (Abcam)) or the respective isotype controls (rabbit IgG isotype control, Invitrogen (Cat. # 02-610-2) and mouse IgG2a kappa isotype control, eBioscience (Cat. # 14-4724-82)) for 30 min at room temperature. Subsequently, the cells were washed three times with buffer A, followed by staining with 5 μg/ml fluorescently labeled secondary antibody (goat anti-rabbit IgG Alexa Fluor 488, Thermo Fisher Scientific, A-11008, or goat anti-mouse IgG (H + L) cross-adsorbed, Alexa Fluor 488, A-11001, Thermo Fisher Scientific) at room temperature for 30 min in the dark. An additional staining using only the secondary antibody was performed to both confirm the absence of nonspecific binding by the secondary antibody and to determine its optimal titer. After three additional washes with buffer A, the stained cells were analyzed using a BD FACSAria Fusion High-Speed Cell Sorter (BD Biosciences) at the UDEL Flow Cytometry Core, Delaware Biotechnology Institute. Flow cytometry analysis was conducted in biological triplicates.

The gating strategy involved selecting cells based on forward (FSC-A) and side (SSC-A) scatter, single cells with forward (FSC-H v FSC-A) singlet events, and mCherry positive events (for multiplicity of infection+, V_1_ viral titer events) in the PE-Texas Red channel (600LP, 610/20BP). A minimum of 100,000 events were acquired for each sample and evaluated on the FITC channel for ABCA4-Alexa488 expression. Data analyses were performed on FlowJo software (Version 10.9.0) (https://www.flowjo.com/). Initially, 10,000 events for each multiplicity of infection+ (mCherry+) population were downsampled using DownsampleV3 on FlowJo, followed by gating for ABCA4-Alexa488+ events on the FITC channel (502LP, 530/30BP). Median fluorescence intensity for ABCA4-Alexa488+ populations was calculated according to median fluorescence in the FITC + positive gates.

### Nanoparticle size determination

The size of the VLPs was determined using the microfluidic resistive pulse sensing method with the Spectradyne nCS1 particle analysis instrument, hardware version 2. Measurements of VLP samples were taken post-qEV column purification, using the 1% tween-20 (v/v) in DPBS microfluidic system for priming. Samples (or buffer B for blank) were diluted (1:1 v/v) in buffer B and loaded into C400 microfluidic cartridges (Spectradyne LLC). Ten seconds of data acquisition was repeated until reaching enough samples for <1% error rate. The analysis was performed in triplicate. Data was combined and processed in the nCS1 Data Viewer software (Version 2.5.0.076) (https://nanoparticleanalyzer.com/dlhidgen3/). Peak filtering, background subtraction, and blank reading CSD (Concentration Spectral Density) file subtraction were applied following the manufacturer's operation manual.

### Retinal-stimulated ATPase assay

To determine the biological activity of the ABCA4 in VLPs, the basal and retinal-stimulated ATPase assay was conducted following a previously described protocol with slight modifications (54). In brief, 500 μM ATP-α-32P in buffer C was combined with purified ABCA4-VLP. All VLP samples were diluted to 1 μg/μl total protein concentration prior to ATPase measurements. Additionally, the protein content of each VLP preparation was verified by SDS-PAGE, followed by Coomassie Brilliant Blue staining, and WBA. This standardization allowed for a direct comparison of ATPase activity between WT and variant ABCA4 proteins. A total of 10 μl reaction mixture with or without 40 μM ATR - 140 μM PE (DOPE) mixture was incubated in the dark at 37 °C for 60 min. The radioactive mixtures were spotted on PEI cellulose plates. The strips were developed in a TLC chamber in 1 M formic acid, 0.5 M LiCl solution. The spots containing the separated ADP and ATP were visualized and excised from the strips under UV fluorescence. The strips were counted using a Beckman 6500 liquid scintillation counter. The obtained data were used to calculate ATPase activity (pmol/min) using GraphPad Prism software (GraphPad Software, Inc) (https://www.graphpad.com/features). All assays were performed in independent biological triplicate and reported with SD.

### *In silico* protein structure analysis

The *in silico* structure analysis of the variant proteins was performed as outlined previously (51) using available experimental structures of human ABCA4 (90, 91, 94) and the refined AlphaFold2 models (96, 97). PyMOL2 software (The PyMOL Molecular Graphics System, Version 2.0 Schrödinger, LLC) (https://www.pymol.org/) was used for protein model visualization and analysis.

In addition, computational pathogenicity prediction for the p.Y1779F VUS was conducted using PolyPhen-2 (http://genetics.bwh.harvard.edu/pph2/) (71), REVEL (https://sites.google.com/site/revelgenomics/) (72), MutationTaster (https://www.mutationtaster.org/) (74), CADD (https://cadd.bihealth.org/snv) (73), and ConSurf (https://consurf.tau.ac.il/consurf_index.php) (98).

### Other methods

Protein concentrations were determined by the Bradford method (95) using bovine serum albumin as a standard.

### Statistical analysis

ATPase activity assays were performed in triplicate for both WT and ABCA4 variants (N965S, C1488R, Y1779F). A one-way ANOVA was used to compare the basal ATPase activities of each variant with the WT. Subsequently, a separate one-way ANOVA was used to compare the fold change in retinal-stimulated ATPase activities. Model residuals were examined for outliers, normality, and variability using externally studentized residuals with 95% simultaneous limits (Bonferroni), individual limits analyses, residual normal quantile plots, and leverage plots and were found consistent with ANOVA assumptions.

## Data availability

The data that support the findings of this study are available from the corresponding author upon reasonable request.

## Supporting information

This article contains supporting information.

## Conflict of interest

The authors declare that they have no conflicts of interest with the contents of this article.
