# Supplementary material for: Virus-like particles as robust tools for functional assessment: Deciphering the pathogenicity of ABCA4 genetic variants of uncertain significance
Source: J Biol Chem. 2024 Aug 31;300(10):107739. doi: 10.1016/j.jbc.2024.107739 (PMC11474199; doi:10.1016/j.jbc.2024.107739)

Virus-like particles as robust tools for functional assessment: Deciphering the pathogenicity of *ABCA4* genetic variants of uncertain significance.

Senem Cevik ^1,2^, Subhasis B. Biswas ^1,2^, Arit Ghosh ^3^, and Esther E. Biswas-Fiss ^1,2^*

^1^ Department of Medical and Molecular Sciences, University of Delaware, College of Health Sciences

^2^ Ammon Pinizzotto Biopharmaceutical Innovation Center, University of Delaware, 590 Avenue 1743, Newark, DE 19713

^3^ Delaware Biotechnology Institute, UD Center for Bioimaging, University of Delaware, Newark, DE

List of the materials:

Table S1. List of primers used in site-directed mutagenesis (SDM).

Table S2. Relative quantification of ABCA4 variants (p.N965S, p.C1488R, and p.Y1779F) in High5 cells and virus-like particles (VLPs) compared to the wild-type (WT) from Western Blot Analysis.

Figure S1. Validation of specificity in flow cytometry analysis using various control experiments.

Figure S2. Enzyme titration ATPase activity of VLP-ABCA4-WT.

Figure S3. Enzyme titration ATPase activity of VLP-ABCA4 variants.

Figure S4. Flow cytometry analysis revealed that the topological orientation of the p.Y1779F variant remained unchanged, exhibiting the same topology as the WT ABCA4 in the cell plasma membrane.

Table S1. List of primers used in site-directed mutagenesis (SDM).

| **Location** | **Pair Of Primers** |
| --- | --- |
| ***ABCA4: c.2894A>G (p.N965S)***  *Chr1:94046943* | *F:5́- CCAGCTCCACTGTGGCCCAGGAATGCG -3́* |
|  | *R:5́- CGCATTCCTGGGCCACAGTGGAGCTGG -3́* |
| ***ABCA4: c.4462T>C (p.C1488R)***  *Chr1:94029522* | *F:5́- GTGCTGCACCTGCGGGATGGTGAAGGG -3́* |
|  | *R:5́- CCCTTCACCATCCCGCAGGTGCAGCAC -3́* |
| ***ABCA4: c.5336A>T (p.Y1779F)***  *Chr1:94014667* | *F:5́- GGATGCTGGGAACATCATGGGAATGACCGC -3́* |
|  | *R:5́- GCGGTCATTCCCATGATGTTCCCAGCATCC -3́* |

Reference genome assembly: GRCh38:Chr1:83457325-104273917, Reference Transcript: M_000350.3.

Table S2. Relative quantification of ABCA4 variants (p.N965S, p.C1488R, and p.Y1779F) in High5 cells and virus-like particles (VLPs) compared to the wild-type (WT) from Western Blot Analysis. The relative quantification was performed using iBright™ Analysis Software (ver. 3.0.1). The data represent the mean expression levels ± standard deviation (SD) from three independent replicates (N=3).

| **Sample** | **Expression level in High5** | | | **VLP-Targeting Level** | | |
| --- | --- | --- | --- | --- | --- | --- |
|  | **Mean** | **SD** | **N** | **Mean** | **SD** | **N** |
| **WT** | 1 | 0 | 3 | 1 | 0 | 3 |
| **p.N965S** | 1.01 | 0.08 | 3 | 0.49 | 0.09 | 3 |
| **p.C1488R** | 1.02 | 0.07 | 3 | 0.98 | 0.12 | 3 |
| **p.Y1779F** | 1.03 | 0.18 | 3 | 1 | 0.06 | 3 |

Figure S1. Validation of specificity in flow cytometry analysis using various control experiments. (A) Flow cytometry analysis of uninfected (left) and ABCA4-VLP-infected (right) Sf9 cells gated for the mCherry-PE-TexasRed positive signal (MOI+) (top) showing no signal in the AF488 channel (bottom), indicating no non-specific signal from unstained controls. (B) Flow cytometry analysis of Sf9 cells infected with negative control baculovirus and stained with Anti-NBD2p, Anti-NBD2m, and Anti-ECD1 primary antibodies followed by AF488-labeled secondary antibody, gated for the mCherry-PE-TexasRed positive signal, showing no signal in the AF488 channel, indicating no non-specific signal from infected cells that do not express ABCA4. (C) Secondary antibody-only controls gated for the mCherry-PE-TexasRed positive signal showing no AF488 signal, confirming the absence of non-specific binding of secondary antibodies. (D) Isotype control experiments of the ABCA4-VLP-infected Sf9 cells gated for the mCherry-PE-TexasRed positive channel showing no AF488 signal, confirming the specificity of the primary antibodies used. Rabbit IgG isotype control is used the validation of Anti-NBD2p and Anti-ECD1 validation, and Mouse IgG2a kappa Isotype Control is used the validation of the Anti-NBD2m antibodies.

**
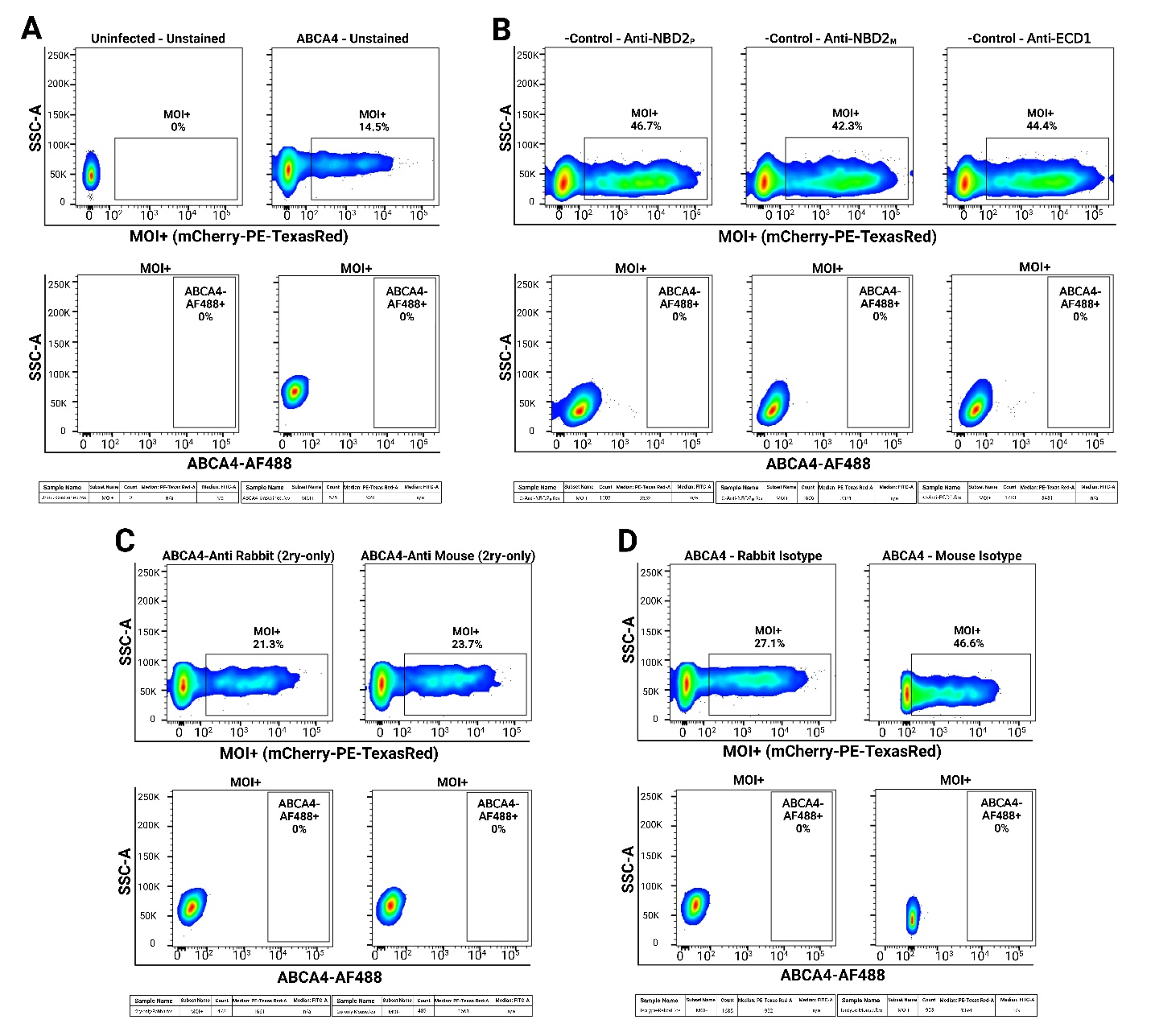
**

**Figure S2.** **Enzyme titration ATPase activity of VLP-ABCA4-WT.** The ATPase assay was conducted at various concentrations to determine the optimal enzyme concentration, where maximum hydrolysis occurs while the activity remains within the linear range. Based on this assay, the data point at 1 µg VLP total protein concentration was selected to compare the variants with the WT. The graph represents the enzymatic activity of VLP-ABCA4-WT protein without background (negative control VLP) subtraction.


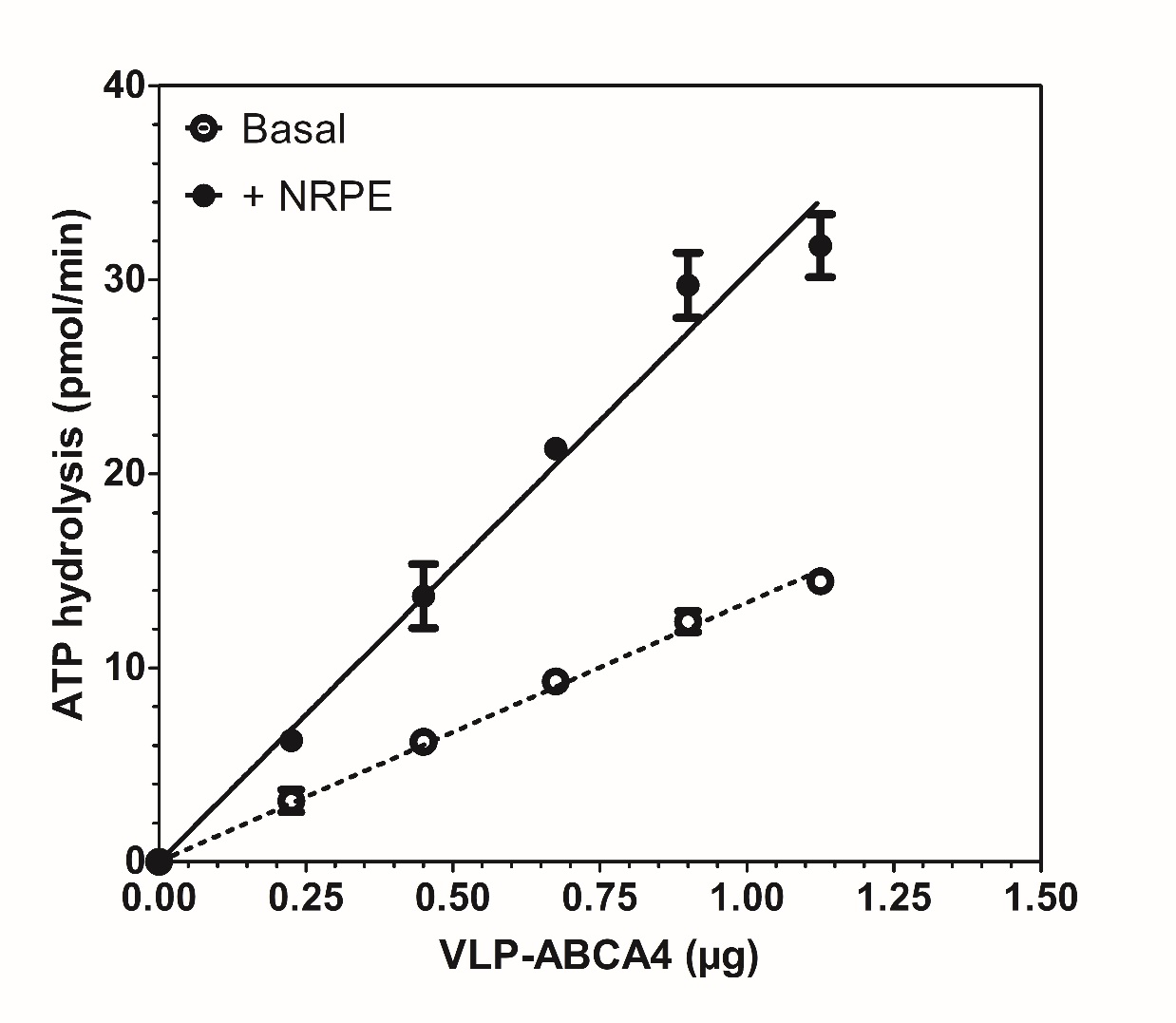


**Figure S3.** **Enzyme titration ATPase activity of VLP-ABCA4 variants.** The ATPase assay was conducted at various concentrations for the variant VLP-ABCA4 variants. The graph represents the enzymatic activity of ABCA4 protein in VLPs after background (negative control VLP) subtraction.


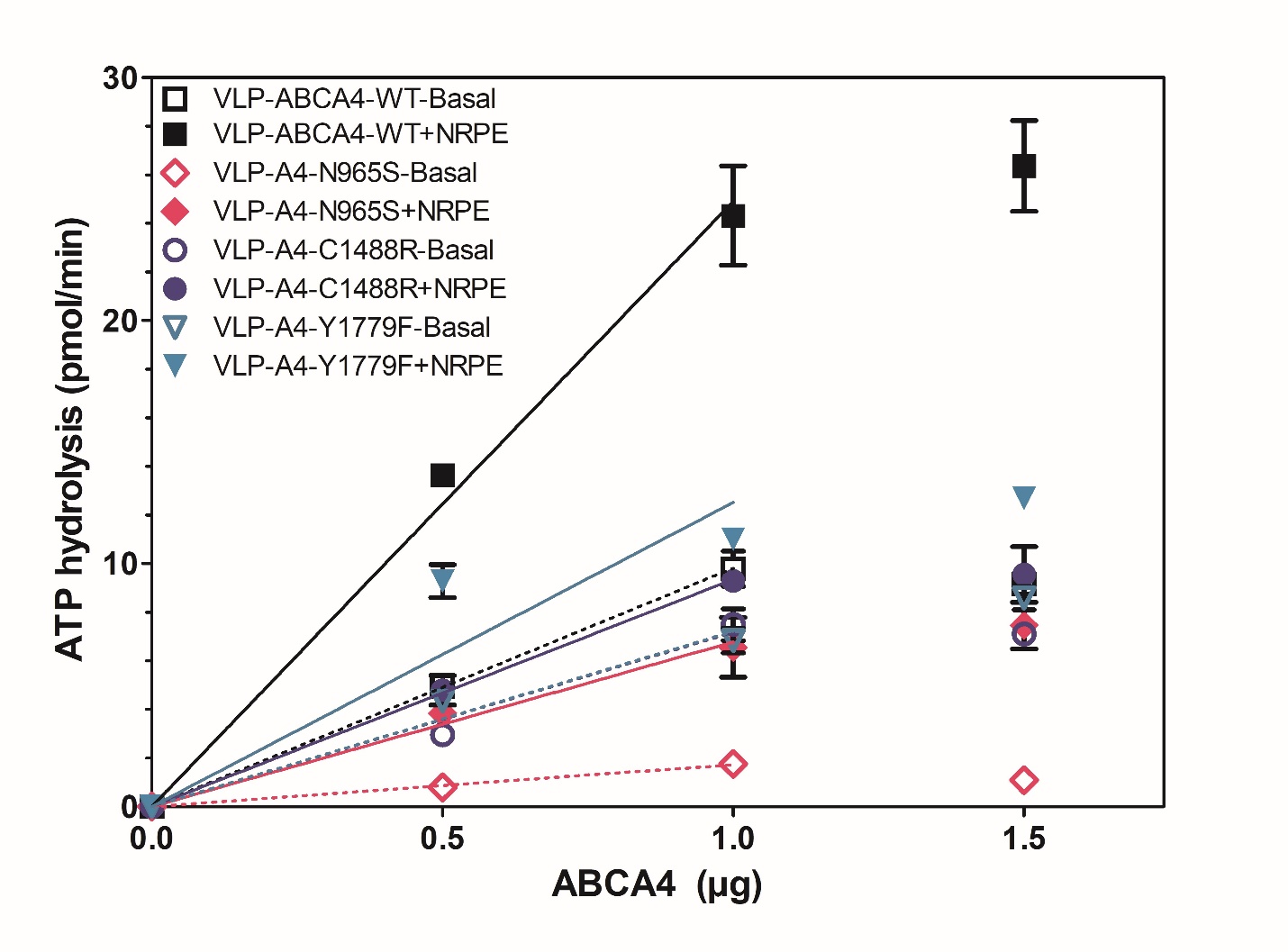


**Figure S4.** **Flow cytometry analysis revealed that the topological orientation of the p.Y1779F variant remained unchanged, exhibiting the same topology as the WT ABCA4 in the cell plasma membrane.** ABCA4-WT and ABCA4-Y1779F expressing cells showed no signal when stained with an ECD1-specific antibody (**A, C**), but displayed a similar level of positive signal when stained with an NBD2-specific antibody (**B, D**). Representative plots are shown from 3 biological replicates. The gating strategy involved selecting cells based on forward (FSC-A) and side (SSC-A) scatter, single cells with forward (FSC-H vs. FSC-A) singlet events, and mCherry positive events (for MOI+) in the PE-Texas Red channel (600LP, 610/20BP). 10,000 events for each MOI+ population were downsampled using DownsampleV3 on FlowJo, followed by gating for ABCA4-Alexa488+ events on the FITC channel (502LP, 530/30BP). Data analyses were performed using FlowJo™ software (Version 10.9.0).


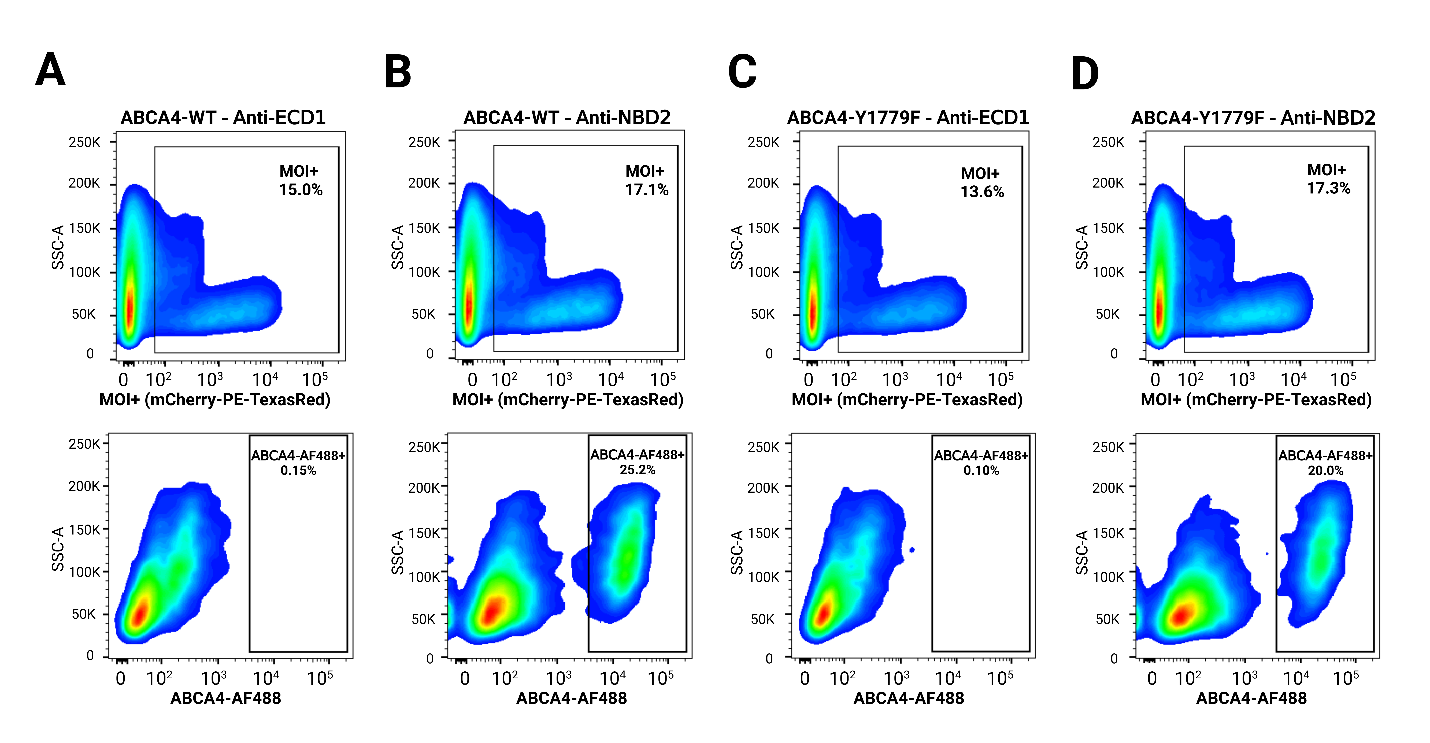

Supplement: Supporting information [file mmc1.docx]
